# An Evaluation of Local Implementing Partner Performance During the First 2 Years of the USAID/PEPFAR Transition

**DOI:** 10.9745/GHSP-D-22-00337

**Published:** 2023-06-21

**Authors:** Lauren A. White, Latham Avery, Lindsay Bonanno, Christy Knight, Courtney Irwin, Kathryn Hoeflich, Deborah Kaliel, Mai Hijazi, E. Callie Raulfs-Wang

**Affiliations:** a Systems & Program Sustainability Division, Office of HIV/AIDS, Bureau for Global Health, U.S. Agency for International Development, Washington, DC, USA.; b Capacity Building and Partnerships Branch, Systems & Program Sustainability Division, Office of HIV/AIDS, Bureau for Global Health, U.S. Agency for International Development, Washington, DC, USA.; c Strategic Information, Evaluation and Informatics Division, Office of HIV/AIDS, Bureau for Global Health, U.S. Agency for International Development, Washington, DC, USA.; d Office of Health Systems, Bureau for Global Health, U.S. Agency for International Development, Washington, DC, USA.

## Abstract

**Introduction:**

Locally led and owned development is considered the best practice for international aid. As an implementing agency for the U.S. President’s Emergency Plan for AIDS Relief (PEPFAR), the U.S. Agency for International Development (USAID) supported the goal of transitioning 70% of its portfolio funding directly to local organizations by 2020, including partner country governments. However, limited evidence or evaluation exists on how such a transition can help achieve HIV-related health outcomes.

**Methods:**

We evaluated monitoring, evaluation, and reporting performance; calculated indicators; and quality of service across the HIV/AIDS treatment cascade for local and international partners in the USAID/PEPFAR portfolio implementing similar programs during the U.S. Government fiscal years (FY) 2019 to 2020 (October 1, 2018–September 30, 2020). We compared results aggregated globally, by country, and across individual partners.

**Results:**

Globally, local partners met a lower proportion of their treatment targets than international partners and did not meet targets for pre-exposure prophylaxis or voluntary medical male circumcision in FY2020. However, local partners exceeded targets in programs supporting orphans, vulnerable children, and key populations affected by HIV/AIDS. Local partners also had testing positivity, linkage rates, and viral load suppression that were equivalent to or higher than that of international partners. Based on available assessments, local partners displayed quality of service delivery comparable to international partners.

**Conclusion:**

Local partners faced challenges, including unfamiliarity with USAID funding, increasing targets across several indicators, and the syndemics of HIV/AIDS and COVID-19. A higher percentage of targets and funding led South African local partners to yield an outsized effect on global percent target achievement. While these findings should be interpreted cautiously due to limited sample size and short time horizon, they are a key first step in evaluating the local partner transition support of the long-term goal of sustained epidemic control of HIV/AIDS.

## Abstract (precis)

We examine local partner performance across the HIV/AIDS clinical cascade during the USAID/PEPFAR transition to local partners.

## Teaser

**Key Findings:**

- Globally, local partners exceeded targets for orphans and vulnerable children and key populations but met a lower proportion of their treatment targets than international partners and did not meet targets for pre-exposure prophylaxis or voluntary medical male circumcision in fiscal year (FY) 2020.
- Local partners had higher mean and median testing positivity, linkage rates, and viral load suppression than international partners, although these differences were not statistically significant.
- Local partners displayed quality of service delivery comparable to international partners across FY2019 and FY2020.

**Key Implications:**

- Sufficient time is needed to implement a responsible transition that supports careful oversight, program monitoring, and capacity development.
- These findings should be interpreted cautiously because of a limited sample size and short time horizon. Continual monitoring of the transition will be critical to sustained progress.
- This preliminary evaluation is a critical step toward increasing country ownership and localization and supporting local partners’ roles in sustained epidemic control of HIV/AIDS.

## INTRODUCTION

Locally led and owned development is considered a best practice for long-term sustainability of development and humanitarian relief programs.^1^ Countries have committed themselves to these priorities through high-level agreements, including the Paris Declaration on Aid Effectiveness in 2005, the Fourth High Level Forum on Aid Effectiveness in Busan in 2011, and the Grand Bargain in 2016. The U.S. Government (USG), including the U.S. Agency for International Development (USAID), has embraced similar objectives through multiple initiatives, such as the Presidential Policy Directive on Global Development,^2^ USAID Forward and Local Solutions,^3^ the Local Systems Framework,^4^ and the Journey to Self-Reliance.

Although increased country ownership and locally led development are commonly cited objectives,^1^^,^^5^^,^^6^ redistributions in funding directly to local actors have been minimal.^7^^,^^8^ While public tracking of local partner funding by USAID has been scarce, in fiscal year (FY) 2012, estimates of the percentage of USAID mission program funds obligated to local organizations in partner countries ranged from 14% to 24%,^9^ and from FY2015 to FY2019, just 25 organizations received half of USAID’s funding, only 1 of which was local.^10^ One potential concern in global health programming is that rapidly expanding direct work with local partners will imperil ongoing service or the ability to swiftly scale up supporting health infrastructure.^5^ “Transition” encapsulates this process of progressing from donor-led to locally led planning, managing, and delivery of health care programs.^11^^,^^12^ However, there are few studies analyzing this critical transition process, including the intermediate steps where donors still provide funding, but local organizations are implementing programs themselves.^11^ Robust evidence on local partner programmatic performance is also scant.^13^^,^^14^

Within the context of HIV/AIDS, a key objective is sustained epidemic control, whereby 95% of people living with HIV (PLHIV) know their status, 95% of those tested are on treatment, and 95% of individuals on treatment are virally suppressed—defined by the 95-95-95 clinical cascade targets established by the Joint United Nations Programme on HIV/AIDS.^15^ A sustainable response to HIV/AIDS requires long-term planning for the transition of donor support to domestic sources that maintain and support sustained epidemic control.^16^^,^^17^ Therefore, the U.S. President’s Emergency Plan for AIDS Relief (PEPFAR) is working to decrease dependence on international implementing partners and scale up reliance on local actors, including national and subnational government entities, nongovernmental organizations, civil society organizations, faith-based organizations, private sector entities, hospitals, pharmacies, and supply chain partners, which together support a country’s health system.

A sustainable response to HIV/AIDS requires long-term planning for the transition of donor support to domestic sources that maintain and support sustained epidemic control.

In 2018, PEPFAR announced the goal of funding 70% of its portfolio directly through local organizations, including partner country governments, by the end of 2020. This objective was built on PEPFAR’s experience in supporting capacity development for local partners since the early transitions of PEPFAR treatment programs.^18^^,^^19^ In FY2018, more than 30% of USAID/PEPFAR program funds went directly to local actors across 23 of its largest countries, with most of that funding occurring in South Africa and Kenya. Directly funding local organizations as part of this transition process is hypothesized to support an effective HIV/AIDS response and sustained epidemic control in several ways^20^: (1) increased local ownership and leadership of local communities and institutions lead to enhanced responsiveness to emerging local needs; (2) legitimacy of programming is increased because it is based on contextually relevant, local expertise at national and subnational levels; (3) a cohort of local organizations and institutions leads to new relationships, communities, and improved information sharing; (4) investments in the financial, procurement, human resource, and governance systems of local organizations strengthen organizational capacity; and (5) working directly with local organizations and institutions with lower overhead may improve cost efficiencies for donors.

From FY2018 to FY2021, PEPFAR-funded USAID programs have steadily increased approved budget amounts to prime local partners, shifting a net amount of US$146 million in direct funding.^20^ In conjunction with this increased level of funding, there has been a commensurate shift in the percentage of programmatic performance targets for which local organizations are responsible—this share has grown by more than 50% since FY2018.^20^ PEPFAR has a standardized process that includes quarterly reviews of monitoring, evaluation, and reporting (MER) targets to routinely measure the performance of individual partners. USAID’s Office of HIV/AIDS has been monitoring local partner performance routinely as part of this standard programmatic oversight and has addressed implementation challenges to these transition goals. In this article, we summarize local partner transition data collected by USAID from implementation years FY2019 to FY2020, with the goal of understanding local partner performance and quality of service delivery during the first 2 years of the local partner transition in response to PEPFAR’s 70% global funding goal.

## METHODS

PEPFAR routinely collects data on all aspects of program implementation. We evaluated: (1) partner achievement against annual PEPFAR MER targets; (2) calculated indicators as metrics of partner performance; and (3) data collected through a quality assurance tool called Site Improvement through Monitoring System (SIMS) to evaluate quality of services and programs at the facility, community, and above site level. The Box reviews key PEPFAR and USG terminology.

For all analyses, we focused on data from the 23 long-term strategy (LTS) countries. Although regional partners were classified as local beginning in FY2021 per country operational plan guidance (Box), for consistency in these analyses, regional partners are classified as local for all years.^21^^–^^24^ All analyses and figure generation were conducted using Tableau version 2020.3 and R version 4.1.1.

BOX Relevant Definitions

**Country operational plan/regional operational plan** (COP/ROP): The COP/ROP guides PEPFAR activities and implementation in a specific country or region and serves as an annual approval of the specific budgets and targets in a country or region.^21^ Further mention of COP is inclusive of COP/ROP, except as specified.

**Implementing partner**: In PEPFAR, implementing partners are organizations (including but not limited to non-governmental organizations, private sector, partner country governments, civil society organizations, faith-based organizations, and education institutions) that implement awards. Prime partners refer to partners that have direct awards with the U.S. Agency for International Development.^21^ There can be only 1 prime partner per implementing mechanism.^21^^,^^22^

**Local partner**: Local partners are incorporated in the country served by the PEPFAR program and either owned or staffed by a majority of citizens or legal residents of that country (the Supplement includes full definitions).^21^

**Regional partners**: Regional partners are incorporated in another country in the region (as classified by the U.S. Department of State) rather than the specific country in which they are implementing (e.g., a partner incorporated in South Africa but operating in Angola).^23^ Beginning in FY2021, PEPFAR classified regional partners as local partners.^21^

**International partners**: International partners are implementing partners that do not meet the requirements to be classified as a local or regional partner.

**To be determined (TBD) awards**: TBD awards apply to situations where USAID has approved funding for a given award mechanism but has not yet identified an implementing partner. TBD awards may be made to either international or local partners.

**Long-term strategy (LTS) countries**: LTS countries were a historic categorization of 23 PEPFAR countries (the Supplement includes the full list) where PEPFAR has engaged heavily in direct HIV service delivery in an effort to mitigate a more generalized HIV/AIDS epidemic.

**Operating units** (OUs): An OU is any country or region in which PEPFAR implements activities.

**Fiscal year** (FY): For ease and clarification, in this article, FY denotes the year of PEPFAR program implementation (Table 1) and aligns with the U.S. Government’s fiscal year, which runs from October 1 to September 30.

**Above-site service delivery**: Above-site activities are those that occur above the facility or community delivering health services. Such activities can include procurement and supply chain development or maintenance, demand generation activities (e.g., media campaigns), laboratory system or health information infrastructure, program management, and oversight or monitoring.^24^

### MER Targets and Performance

PEPFAR measures partner performance against quantitative targets assigned to specific partners and captured by the MER indicators.^21^ The setting of targets is a key deliverable of country operational plan development, and the process to finalize targets is iterative and includes multiple country or regional stakeholders and PEPFAR implementing agencies. The first step to target setting entails host governments and the USG agreeing on estimates of PLHIV at a national level. Typically, the Joint United Nations Programme on HIV/AIDS leads a process to model the estimates with various epidemiologic data inputs, including population-based survey data and previous estimates, among others. Once these national-level estimates of PLHIV are agreed to, national-level targets are decided to ensure progress to epidemic control; those targets are then allocated down to subnational units and the implementing partners. The U.S. Department of State’s Office of the Global AIDS Coordinator and Health Diplomacy approves the final targets, which are agreed upon by implementing partners. PEPFAR implementing partners self-report on their performance on MER indicators via the Data for Accountability, Transparency, and Impact database—PEPFAR’s health information platform for managing MER, expenditure, and epidemiological data.

We assessed partner performance across 7 MER indicators corresponding to key PEPFAR program areas for treatment, prevention, and support. We examined achievement data for FY2019 and FY2020 implementation (Table 1). Note that for performance, there were no “to be determined” awards included when reporting achievement data. Data are current as of FY2021 Quarter 4 (the period ending September 30, 2021). Final clean data were accessed and exported on January 14, 2022.

**TABLE 1.** Relationship Between Fiscal Year of Implementation, Dates of Program Implementation, and PEPFAR COP Cycle

**Fiscal Year**	**Dates of PEPFAR Program Implementation**	**COP Cycle**
2019	Oct. 1, 2018–Sept. 30, 2019	2018
2020	Oct. 1, 2019–Sept. 30, 2020	2019
2021	Oct. 1, 2020–Sept. 30, 2021	2020

Abbreviations: COP, country operational plan; PEPFAR, U.S. President’s Emergency Plan for AIDS Relief.

We assessed partner performance across 7 MER indicators corresponding to key PEPFAR program areas for treatment, prevention, and support.

For treatment indicators, we evaluated the number of clients testing positive, the number of clients beginning treatment, and the number of clients currently receiving treatment. All 3 indicators are reported quarterly.^25^

For prevention and support indicators, we evaluated the number of individuals served by orphans and vulnerable children (OVC) programs for children and families affected by HIV; the number of key populations (KPs) reached with individual and/or small group-level HIV prevention interventions; the number of individuals who were newly enrolled on oral pre-exposure prophylaxis (PrEP) to prevent HIV infection; and the number of males circumcised through the voluntary medical male circumcision (VMMC) program (the Supplement lists full definitions). The number of individuals who were newly enrolled on oral PrEP and number of males circumcised through the VMMC program are reported quarterly, and individuals served by orphans and vulnerable children (OVC) programs for children and families affected by HIV and KPs reached with individual and/or small group-level HIV prevention interventions are reported semiannually.^25^

### Calculated Indicators: Testing Yield, Linkage to Treatment, and Viral Load Coverage and Suppression

Calculated indicators—including testing yield, linkage to treatment, viral load coverage (VLC), and viral load suppression (VLS)—are another way to assess the performance of partners along the treatment cascade. Testing yield describes the percentage of positives detected out of the total number of clients who were tested and received their results. Linkage to treatment is the percentage of clients who initiate treatment after testing positive in the given reporting period (note: this is reported in aggregate, not at the individual patient level). VLC describes the proportion of eligible patients on antiretroviral therapy (ART) who received a viral load test. Finally, VLS describes the percentage of patients currently on ART who received a viral load test within the last year and have a suppressed viral load (<1000 copies/ml) (full definitions are available in the Supplement).

The expected thresholds for these calculated indicators are at least 95% for linkage to treatment and VLS and at least 80% for VLC.^21^ The expected testing yield depends on the operating units (OUs) and the status of epidemic control since different testing programs and modalities provide variable yields. For these calculations, we only included partners reporting on the number of clients currently receiving treatment (i.e., those partners providing treatment services).

### Service Quality Data

PEPFAR conducts regular assessments of the quality of services and programs at the facility, community, and above site levels through the SIMS tool (Box).^26^ SIMS evaluates HIV core essential elements (CEEs) related to service and non-service delivery functions in different technical areas. USG staff conduct SIMS visits to a chosen sample of PEPFAR sites.

SIMS 4.0, the most up-to-date SIMS assessment, was released in January 2019, so SIMS assessment data began in FY2019, Quarter 2 (i.e., January 1, 2019). We assessed SIMS metrics that roughly correspond to the treatment and prevention categories of interest previously described, in particular: (1) HIV testing; (2) care and treatment; and (3) programming for adolescent girls and young women, gender-based violence, and OVC. The Supplement includes additional information about which CEEs inform these categories.

### Statistical Analyses

We compared the percent achievement of the MER and calculated indicators described above at the OU and individual partner level for international and local partners. We conducted statistical comparisons of local and international partners for each combination of metric and FY (e.g., FY2019 number of clients currently receiving treatment for international vs. local partners). Because sample sizes at the OU and individual partner level were small (often n<30) and the distributions were generally non-normal (as evaluated by QQ-plots), we used a 2-sided Wilcoxon rank sum test with continuity correction where the null hypothesis is that the distributions and medians of the 2 populations are the same.^27^^,^^28^ Statistical significance was evaluated at the *P*=.05 level.

## RESULTS

### Partner Geographic Distribution and Funding Amounts

**FIGURE 1:**
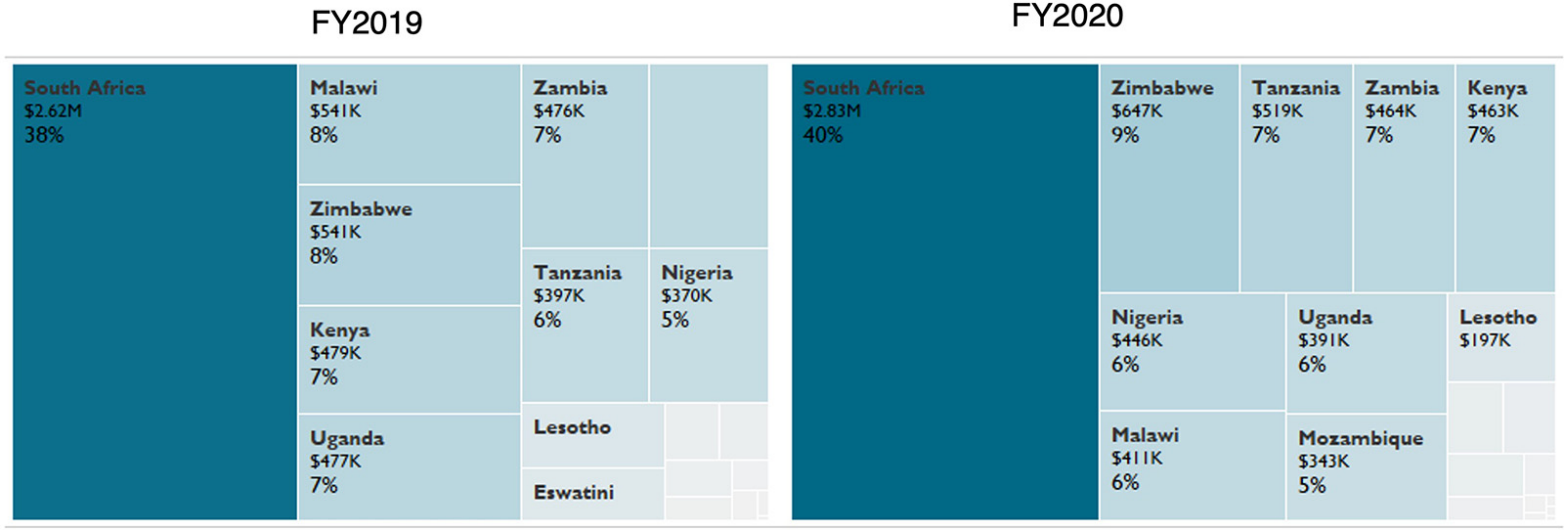
USAID Targets for Number of Clients Currently Receiving Treatment by Operating Unit^a^ Abbreviations: FY, fiscal year; USAID, U.S. Agency for International Development. ^a^USAID target volume and percent target share are displayed for each operating unit for each FY. Cell size and shading is in relation to the proportion of each operating unit currently receiving treatment targets.

The number of local and international partners varies substantially across OUs (Table 2). Between FY2019 and FY2020, the number of local partners across all OUs increased by 46% from 84 to 123, and the total amount of direct funding to local partners increased by 12% from US$580 million to US$648 million (Table 2). South Africa leads in both the number of local partners (n=17 in FY2020) and the total amount of local partner funding for a single OU: $288 million in FY2020 or roughly 45% of the total direct funding for local partners across all OUs (Table 2). This heterogeneity in local partner numbers and funding is also reflected by the numbers of apportioned targets. For example, for currently on treatment targets, South Africa made up 38% and 40% of the target share in FY2019 and FY2020, respectively. The next highest proportions were 8% for Malawi in FY2019 and 9% for Zimbabwe in FY2020 (Figure 1).

**TABLE 2.** Local Partners and International Partner Numbers and Funding Comparison by Operating Unit^a^

**Operating Unit**	**FY2019**				**FY2020**			
	**Number of International Partners**	**Funding to International Partners, US$**	**Number of Local Partners**	**Total Funding to Local Partners by OU, US$**	**Number of International Partners**	**Funding to International Partners, US$**	**Number of Local Partners**	**Total Funding to Local Partners by OU, US$**
Botswana	8	23,637,384	0	0	2	10,290,368	3	700,000
Burundi	3	9,535,632	1	110,929	5	9,590,709	3	1,177,546
Cameroon	2	6,214,748	0	0	4	13,332,872	0	0
Côte d'Ivoire	8	23,174,946	3	3,212,320	7	23,530,865	4	2,142,942
DRC	5	19,538,684	0	0	6	18,633,725	1	2,400,000
Eswatini	10	25,584,903	5	8,503,646	11	26,283,281	3	5,847,954
Ethiopia	7	22,017,851	8	3,207,050	6	15,787,006	12	11,847,621
Haiti	8	18,528,541	1	2,200,000	8	15,853,339	5	7,037,430
Kenya	14	103,581,403	8	133,980,064	13	80,578,056	7	105,366,991
Lesotho	7	37,016,555	3	3,850,732	6	29,256,327	3	11,979,158
Malawi	12	35,551,723	5	20,982,663	10	34,031,457	8	27,731,056
Mozambique	19	92,491,777	13	7,869,552	13	71,283,975	16	9,040,109
Namibia	7	15,492,415	4	11,781,373	9	10,068,903	4	15,119,769
Nigeria	7	38,755,169	3	8,128,787	9	42,342,982	6	27,911,182
Rwanda	1	5,665,423	4	3,915,012	3	3,906,480	4	7,082,826
South Africa	13	66,625,895	20	280,997,977	10	76,523,017	17	288,137,915
South Sudan	3	3,610,094	0	0	3	4,265,926	0	0
Tanzania	17	129,991,304	5	21,810,908	17	91,137,797	12	36,906,819
Uganda	24	98,607,150	3	1,957,453	26	87,763,888	10	5,937,831
Ukraine	3	5,250,000	1	4,321,558	3	4,598,049	1	4,800,000
Vietnam	4	9,216,382	3	1,879,740	3	9,062,511	3	2,402,103
Zambia	11	79,632,160	5	37,563,776	8	81,888,319	6	48,141,202
Zimbabwe	4	37,713,806	6	23,902,597	5	37,371,048	5	25,986,378
Grand Total	74	907,433,945	84	580,176,137	65	797,380,900	123	647,696,832

Abbreviations: DRC, Democratic Republic of the Congo; LTS, long-term strategy; OU, operating unit; TBD, to be determined; US$, U.S. dollars.

^a^ Columns sum to a greater number than is listed in the “grand total” row because the OU rows sum the number of unique partners in each OU, but the grand total row does not double count a single partner working in multiple OUs. For example, if Company X is working in both Botswana, Burundi, and Cameroon, it will be counted as 1 unique partner each of their respective rows, which would sum to 3. However, the grand total column will only count 1 instance of Company X across all OUs. Totals include LTS OUs only, and regional partners counted as local. TBD awards were excluded from funding calculations.

### Globally Aggregated Performance

**FIGURE 2:**
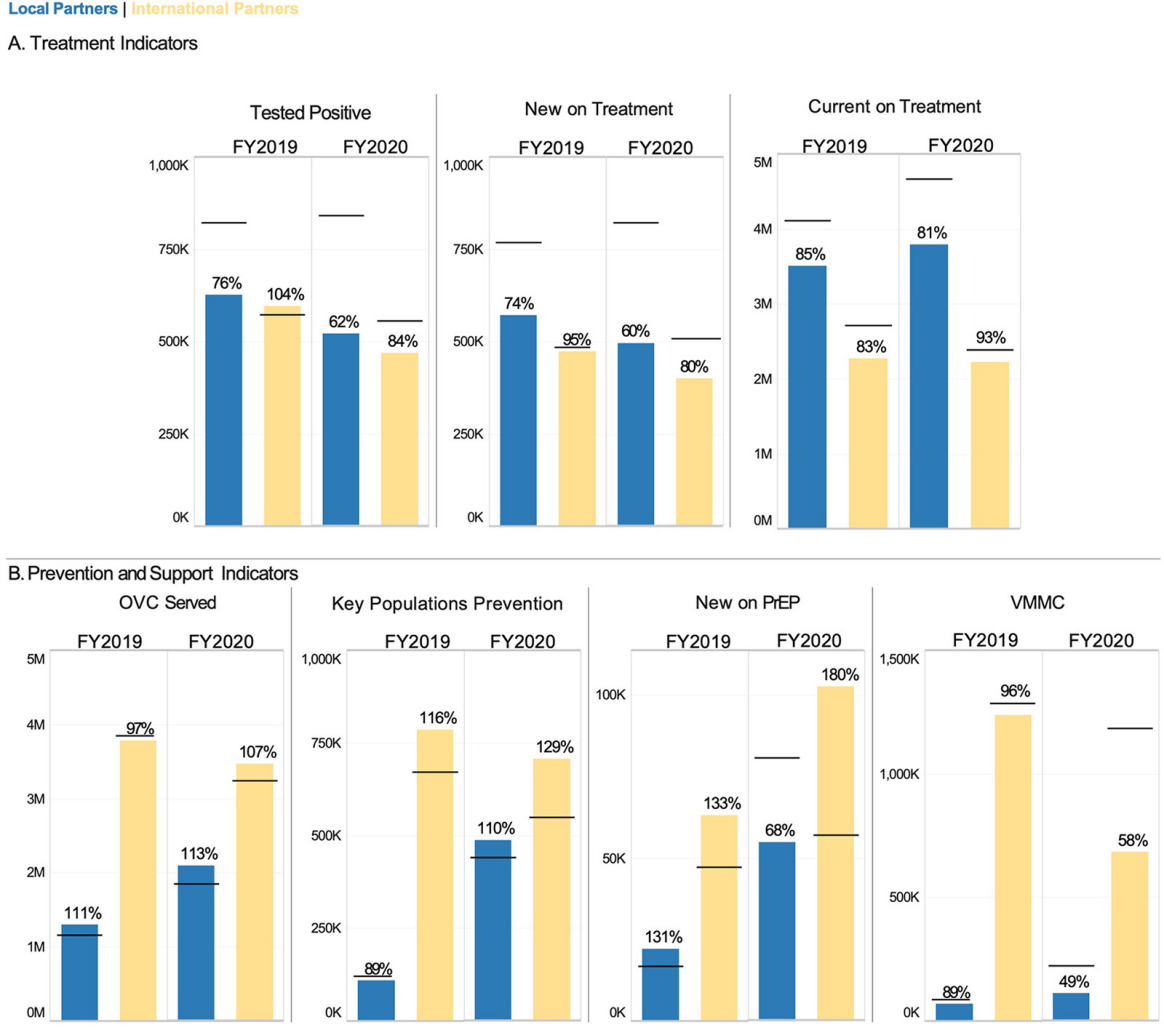
Globally Aggregated Achievement of Programmatic Targets in Key Technical Areas of (A) Treatment and (B) Prevention and Support for USAID/PEPFAR International vs. Local Partners Across 23 LTS Country Programs^a^ Abbreviations: FY, fiscal year; OVC, orphans and vulnerable children; PEPFAR, U.S. President’s Emergency Plan for AIDS Relief; LTS, long-term strategy; PrEP, pre-exposure prophylaxis; USAID, U.S. Agency for International Development; VMMC, voluntary medical male circumcision. ^a^The horizontal line corresponding to each bar indicates the target set before implementation. The percentage over each bar indicates the percent target achievement during that implementation year. The y-axis for each indicator is scaled to the total number of targets to be achieved.

**FIGURE 3:**
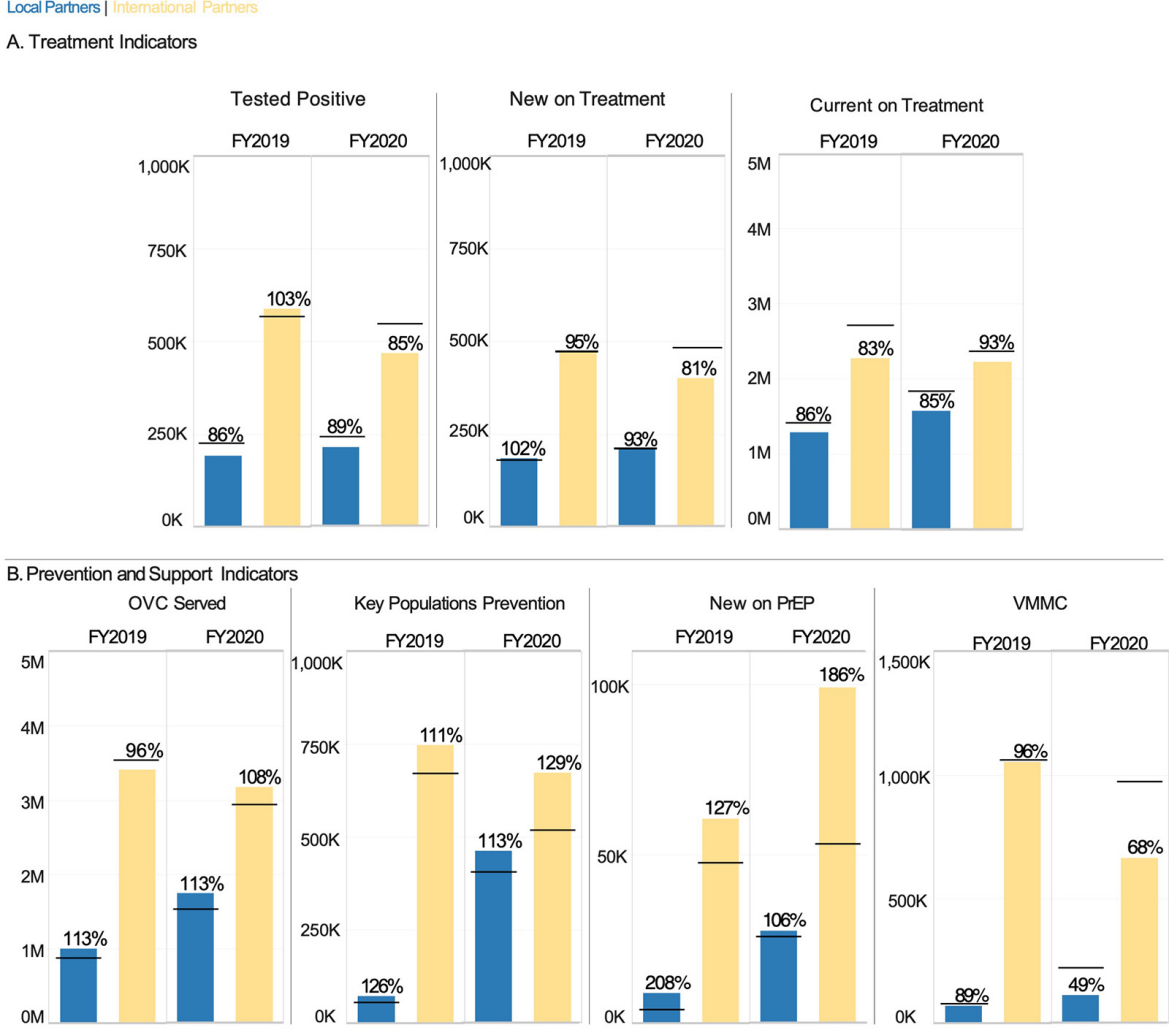
Achievement of Programmatic Targets in Key Technical Areas of (A) Treatment and (B) Prevention and Support for USAID/PEPFAR International vs. Local Partners Across 22 LTS Country Programs Excluding South Africa^a^ Abbreviations: FY, fiscal year; PEPFAR, U.S. President’s Emergency Plan for AIDS Relief; LTS, long-term strategy; USAID, U.S. Agency for International Development. ^a^The horizontal line corresponding to each bar indicates the target set before implementation. The percentage over each bar indicates the percent target achievement during that implementation year. The y-axis for each indicator is scaled to the total number of targets to be achieved.

**FIGURE 4:**
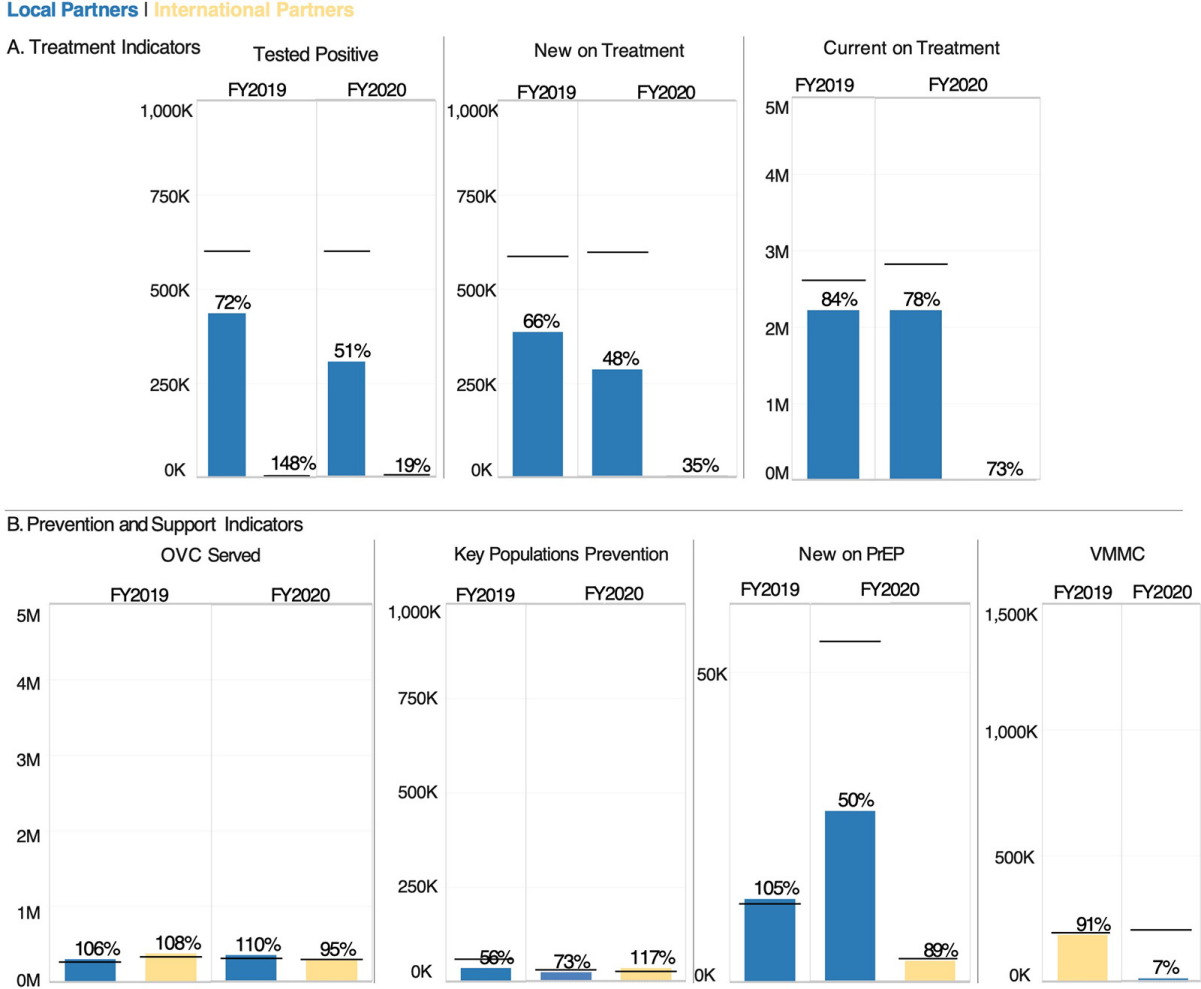
Achievement of Programmatic Targets in Key Technical Areas of (A) Treatment and (B) Prevention and Support for USAID/PEPFAR International vs. Local Partners in South Africa Abbreviations: FY, fiscal year; OVC, orphans and vulnerable children; PEPFAR, U.S. President’s Emergency Plan for AIDS Relief; LTS, long-term strategy; PrEP, pre-exposure prophylaxis; USAID, U.S. Agency for International Development; VMMC, voluntary medical male circumcision. ^a^The horizontal line corresponding to each bar indicates the target set before implementation. The percentage over each bar indicates the percent target achievement during that implementation year. The y-axis for each indicator is scaled to the total number of targets to be achieved.

Globally, neither international partners nor local partners met 100% of their assigned treatment targets in FY2019 or FY2020, except for international partners’ testing positive target in FY2019 (Figure 2A). In FY2020, local partners reached 62% of testing positive targets, 60% of new on treatment targets, and 81% of their assigned currently on treatment targets, while international partners reached 84%, 80%, and 93%, respectively (Figure 2A). For both international and local partners, there was a consistent drop in performance against targets in testing positive and new on treatment between FY2019 and FY2020. In 22 of the 23 LTS OUs, local partners increased or maintained performance in FY2020 across testing positive (89%), currently on treatment (93%), and new on treatment (85%) (Figure 3). South African local partners, with a large share of global treatment targets, reached 51%, 48%, and 78% of those targets, respectively (Figure 4).

Globally, neither international partners nor local partners met 100% of their assigned treatment targets in FY2019 or FY2020, except for international partners’ testing positive target in FY2019.

For prevention and support, local partners exceeded their OVC served targets (111% in FY2019 and 113% in FY2020), and local partners have also demonstrated strong performance in KPs prevention (89% in FY2019 and 110% in FY2020) (Figure 2B). Local partners had high achievement for new on PrEP and voluntary male medical circumcision in FY2019 (131% and 89%, respectively), but these percent achievements decreased in FY2020 to 68% and 49%, respectively (Figure 2B). International partners were able to maintain performance in new on PrEP (133% in FY2019 and 180% in FY2020), KPs prevention (116% in FY2019 and 129% in FY2020), and OVC served (97% in FY2019 and 107% in FY2020). However, performance in voluntary male medical circumcision declined from 96% in FY2019 to 58% in FY2020 (Figure 2B). As for treatment targets, local partners outside of South Africa were able to maintain new on PrEP achievement in FY2020 at 106% (Figure 3). Local partners in South Africa exceeded targets for OVC served in FY2019 and FY2020, KPs prevention in FY2020, and new on PrEP in FY2020 (Figure 4). For FY2020, the new on PrEP target share tripled for South African local partners, so percent achievement still decreased, even though the number of new on PrEP individuals doubled between FY2019 and FY2020 (Figure 4).

### OU and Individual Partner Level Performance

**FIGURE 5:**
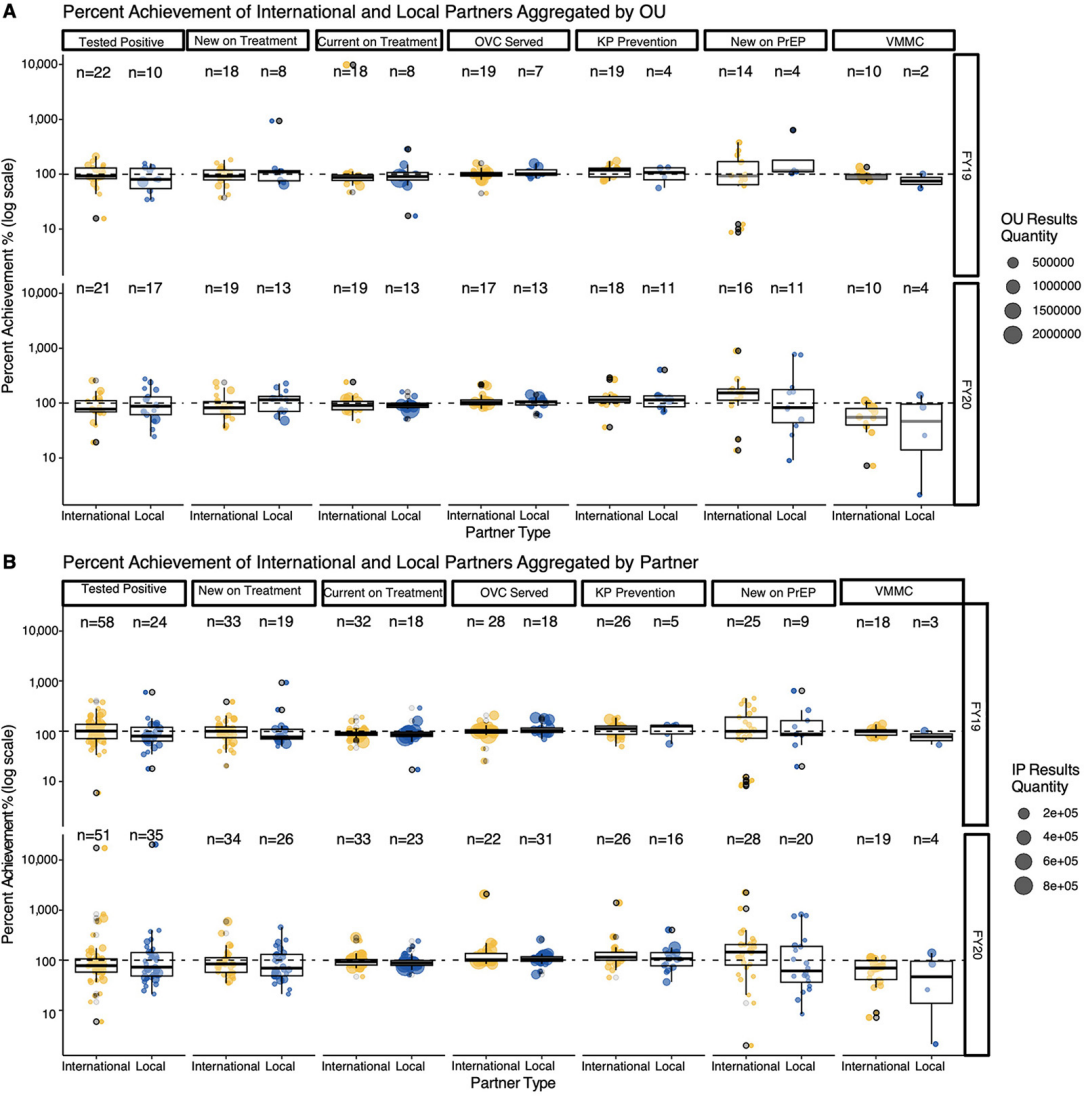
Achievement of Targets Aggregated at the (A) Operating Unit Level or (B) Individual Partner Level for International and Local Partners^a^ Abbreviations: KP, key populations; OU, operating unit; OVC, orphans and vulnerable children; PrEP, pre-exposure prophylaxis; VMMC, voluntary medical male circumcision. ^a^Percent achievement is displayed on a log scale. The first and third quartiles (the 25th and 75th percentiles) are displayed as the lower and upper hinges. Each whisker extends from the hinge to the highest or lowest value no further than 1.5 times the interquartile range from the respective hinge. Individual OU or partner percent achievements are shown as lighter, jittered points with the size of the jittered circle corresponding to the raw results achieved by each OU or implementing partner, respectively.

While both local and international partners have performance outliers, there was not a statistically significant difference in the performance distributions between international and local partner groups for FY2019 and FY2020 when aggregated by OU (Figure 5A; Supplement Table S1: 2-sided Wilcoxon rank sum tests) or by individual partner (Figure 5B; Supplement Table S2: 2-sided Wilcoxon rank sum tests) across the 7 MER indicators. The variation in performance was generally greater for individual partners (Figure 5B) than for OU-level performance (Figure 5A). Cumulative indicators like OVC served and currently on treatment generally had less heterogeneity across OUs or individual partners. For FY2020, local partner performance was substantially more variable than international partner performance for new on PrEP and VMMC (Figure 2B).

### Calculated Treatment Program Indicators: Testing Yield, Linkage to Treatment, VLC, and VLS

From FY2019 to FY2020, local partners reported higher globally aggregated percentages of linkage to treatment and higher VLS than international partners (Table 3). For FY2019, local partners had a higher globally aggregated testing yield percentage, but in FY2020, international and local partners had equivalent testing yields. Local partners had slightly higher VLC than international partners in FY2019 but lower VLC in FY2020 (Table 3). Neither local nor international partners reached the PEPFAR threshold of 95% linkage to treatment or VLS or 80% threshold for VLC.

**TABLE 3.** Globally Aggregated Testing Yield, Linkage to Treatment, Viral Load Coverage, and Viral Load Suppression for International and Local Partners in FY2019 and FY2020^a^

	**International Partners**		**Local Partners**	
	**FY2019**	**FY2020**	**FY2019**	**FY2020**
Testing yield (no. of HIV-positive test results/ total no. of HIV tests)	3.8% (583,166/1,5217,356)	4.5% (459,375/1,0284,268)	4.5% (627,857/1,3860,152)	4.5% (520,299/11,645,984)
Linkage to treatment (no. of clients with HIV-positive test results initiating treatment/ total no. of HIV+ tests)	79.2% (462,159/5,831,660)	85.8% (394,243/459,375)	91.0% (571,543/627,857)	95.2% (495,410/520,299)
Viral load coverage (no. of ART patients with a VL result within the last year/no. of patients on treatment 6 months prior)	78.3% (1,559,602/1,991,610)	77.5% (1,642,612/2,118,905)	80.1% (2,470,359/3,084,502)	71.2% (2,657,202/3,732,035)
Viral load suppression (no. patients on ART who received a viral load test and are virally suppressed/ no. of patients currently on ART who received a viral load test)	88.6% (1,382,535/1,559,602)	91.6% (1,505,228/1,642,612)	91.7% (2,264,823/2,470,359)	92.8% (2,466,371/2,657,202)

Abbreviation: ART, antiretroviral therapy; FY, fiscal year; VL, viral load.

^a^These statistics only include partners reporting those currently on treatment (i.e., those partners providing treatment services). The table includes both the calculated indicators (%) and the underlying raw numbers for each calculation. Regional partners are counted as local in both FY2019 and FY2020. To be determined awards are excluded from calculated indicator calculations.

**FIGURE 6:**
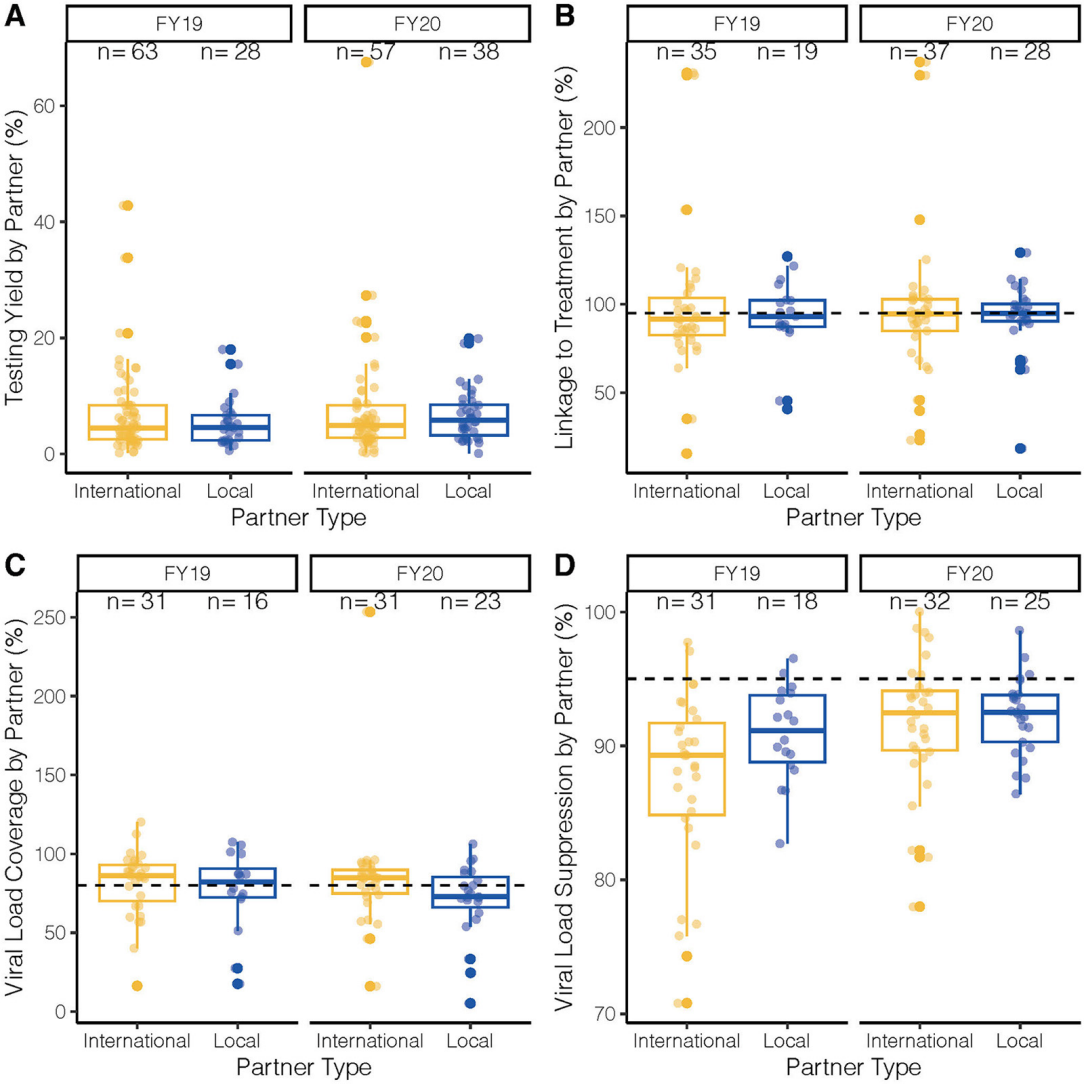
Calculated Indicators of Individual Partners Disaggregated by FY and Partner Type: (A) Test Positivity, (B) Linkage to Treatment, (C) Viral Load Coverage, and (D) Viral Load Suppression^a^ Abbreviation: FY, fiscal year. ^a^The first and third quartiles (the 25th and 75th percentiles) are displayed as the lower and upper hinges. Each whisker extends from the hinge to the highest or lowest value no further than 1.5 times the interquartile range from the respective hinge. Individual partner linkages are shown as lighter, jittered points. The dashed line corresponds to target thresholds for these indicators: 95% for linkage and viral load suppression, 80% for viral load coverage. Y-axes are scaled differently for each indicator. Only partners reporting both number of clients testing positive and number of clients currently receiving treatment were included for linkage calculations. Regional partners were classified as local across both fiscal years.

Individual partner testing positivity did not differ significantly in FY2019 or FY2020 (Figure 6A; Supplement Table S3). When comparing individual partner performance in linkage rates, international partners had a median linkage rate of 91.6%, and local partners had a median linkage rate of 93.2% in FY2020 (Figure 6B; Supplement Table S3). In FY2019, international and local partners had median linkage rates of 94.5% and 94.9%, respectively (Figure 6B; Supplement Table S3). Linkage rates of international vs. local partners did not differ significantly in either FY2019 (Wilcoxon rank sum exact test: W=368, *P*=.53) or FY2020 (Wilcoxon rank sum exact test: W=548, *P*=.70). For VLC and VLS, international and local partner distributions did not differ significantly (Figure 6C and 6D; Supplement Table S3), although this difference approached significance for VLC in FY2020 (median international VLC: 84.8% and median local partner VLC: 72.8%; Wilcoxon rank sum exact test: W=458.5, *P*=.08) and for VLS in FY2019 (median international VLS: 89.3% and median local partner VLS: 91.2%; Wilcoxon rank sum exact test: W=193; *P*=.08).

### Service Quality

**FIGURE 7:**
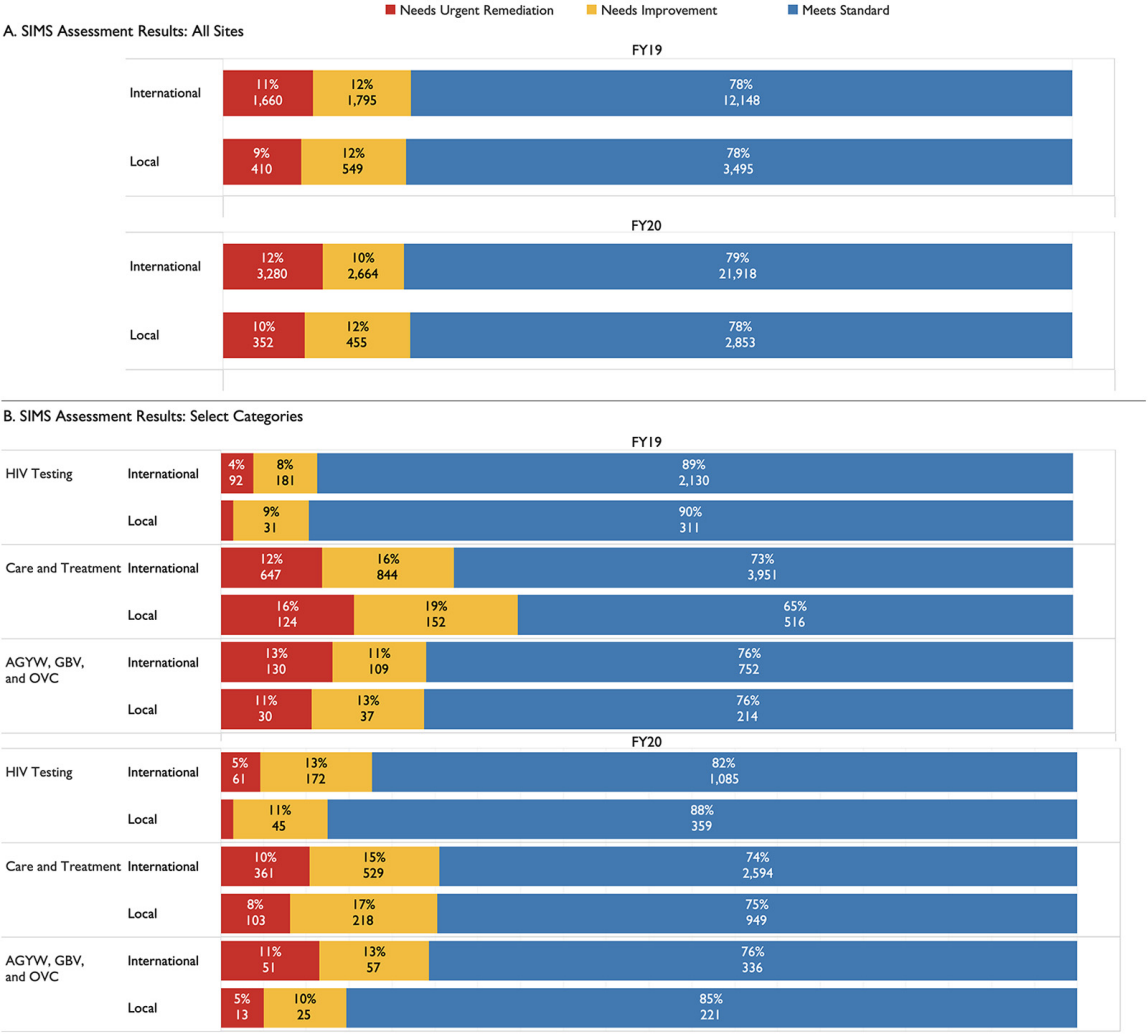
Service Quality of USAID/PEPFAR International vs. Local Partners (A) Across All Sites and (B) by Select SIMS Categories^a^ Abbreviations: AGYW, adolescent girls and young women; CEE, core essential element; FY, fiscal year; GBV, gender-based violence; MER, monitoring, evaluation, and reporting; OVC, orphans and vulnerable children; PEPFAR, U.S. President’s Emergency Plan for AIDS Relief; SIMS, Site Improvement Through Monitoring System. ^a^The scores on SIMS are coded as “needs urgent remediation” (Red), “needs improvement” (Yellow), and “meets standard” (Green). Numbers under the percentages in each category respond to CEE scores, not to the number of site assessments; multiple CEE scores are assessed per each site visit. Of note, SIMS CEEs measure quality metrics at a facility, community, or above site level, while MER indicators are measures of outputs at a partner level, thus MER indicators do not align exactly with SIMS categories but may inform multiple CEEs.^29^ The specific CEEs assessed for each SIMS category are available in the . Current as of June 2021.

In FY2019, 889 SIMS assessments were conducted across 23 LTS OUs, 147 of which were for local partners (Table 4). In FY2020, 567 assessments were conducted, 190 of which were for local partners (Table 4). The proportion of SIMS site visits did not align proportionally with OU-level or partner-level targets: only 17% and 33% of the SIMS site assessments were conducted among local partners in FY2019 and FY2020, respectively (Table 4). The number of SIMS site assessments was also not proportional to the OU-level treatment targets (Figure 1; Supplement Figure). For example, site visits were overrepresented for international partners in Nigeria in FY2019 and for local partners in Namibia in FY2020. For the assessments conducted, local partners showed quality of services comparable to international partners across FY2019 and FY2020 (Figure 7A)^29^ and across different SIMS technical categories, with the exception of care and treatment in FY2019 (Figure 7B).

**TABLE 4.** Number of SIMS Visits and CEEs Assessed for Local and International Partners in FY2019 and FY2020

**Site-Level Assessments**	**FY2019**			**FY2020**		
	**Total**	**Local Partners**	**International Partners**	**Total**	**Local Partners**	**International Partners**
Total SIMS visits	889	147	742	567	190	377
Total CEEs assessed	31,522	3,578	27,944	20,057	3,857	161,200

Abbreviations: CEE, core essential elements; FY, fiscal year; SIMS, Site Improvement Through Monitoring System.

## DISCUSSION

The USAID/PEPFAR local partner transition supports the broader goal of achieving sustained epidemic control of HIV/AIDS following the HIV treatment cascade that consists of: identifying PLHIV, linking PLHIV to treatment and maintaining them on ART, and the final goal of VLS. Performance, calculated indicators, and quality of clinical service delivery provide different viewpoints for assessing these outcomes and their impact on the treatment cascade and prevention. For the first stage of identifying PLHIV (number of clients testing positive), at a global scale, local partners achieved a lower target achievement compared to international partners in FY2019 (76%) and FY2020 (62%) (Figure 2A). This may have been attributable to a decrease in percent achievement for South African local partners from 72% to 51% between FY2019 and FY2020 (Figure 4) since local partners in OUs outside of South Africa slightly increased their percent achievement from 86% to 89% between FY2019 and FY2020 (Figure 3). At an individual partner level, local partners had a lower median percent achievement than international partners in FY2019 (local: 79.7% vs. international: 101.3%) and FY2020 (local: 72.7% vs. international: 77.8%) (Supplement Table S2). However, local partners showed no differences in testing yield (Table 3; Figure 6A; Supplement Table S3) or service quality delivery as indicated by SIMS CEEs related to HIV testing (Figure 7B).

For the first stage of identifying the number of clients testing positive, at a global scale, local partners achieved a lower target achievement compared to international partners in FY2019 and FY2020.

For the next step of the treatment cascade of linking PLHIV to treatment and maintaining them on ART, the global target share for new on treatment and currently on treatment increased for local partners between FY2019 and FY2020,^20^ percent achievement decreased, and was lower than for international partners (Figure 2A). As previously noted, local partners in the 22 LTS OUs outside of South Africa were able to maintain or increase their performance across treatment indicators in FY2020 (Figure 3A). When aggregated by OU, local partners had a slightly higher median percent achievement for currently on treatment and new on treatment for both years, although neither of these comparisons with international partners was statistically significant (Figure 5A; Supplement Table S1). South Africa’s outsized impact on local partner performance, with the largest currently on treatment targets and the largest local partner portfolio for USAID/PEPFAR (Figure 1; Table 2), reflects the challenges of working in a country with the largest HIV seropositive population in the world,^30^ along with COVID-19 lockdowns in 2020. For example, clinic-based estimates in South Africa suggest that HIV testing decreased by nearly 50% during South Africa’s lockdown in April 2020.^31^ Most South African partners are local, which points to the relative success of newly established local partners in other OUs, as well as the importance of monitoring and support to existing local partners.

Globally, local partners were effective at linking newly diagnosed PLHIV to treatment (Table 3) and showed no differences at the individual partner level in linkage to treatment rates (Figure 6B; Supplement Table S3). Despite COVID-19 limiting attendance at health facilities, partners were able to maintain the size of their treatment cohorts (Figure 2A), possibly through an increased focus on differentiated service delivery.^32^ SIMS data corroborate these results, as local partners had comparable findings to international partners and reduced the number of sites that required urgent remediation in the area of Care and Treatment between FY2019 to FY2020 (Figure 7B).

The final step of the clinical cascade is VLS, which is critical for interrupting the transmission cycle since PLHIV on treatment with an undetectable viral load have effectively no risk of sexually transmitting HIV.^33^ VLC reflects how much of the eligible patient population is receiving viral load testing. Local and international partners reported roughly equivalent rates of globally aggregated VLC of approximately 70%–80%, with local partners performing slightly better in FY2019 and international partners performing slightly better in FY2020 (Table 3). These global performance trends were also reflected in median partner VLC, although those differences were not statistically significant (although approaching significance in FY2020) (Figure 6C; Supplement Table S3). Although neither local nor international partners achieved the Joint United Nations Programme on HIV/AIDS target of 95% for VLS, local partners reported slightly higher rates of VLS compared to international partners both globally (Table 3) and individually (Figure 6D; Supplement Table S3), but these differences were not significant at the individual partner level.

In addition to the clinical cascade, local partners are also expected to implement effective prevention and support programs. Local partner OVC and KP programs reported strong results in both FY2019 and FY2020 (Figure 2B). At an individual partner level, local partners had higher mean and median percent achievement in these 2 areas for both years, although there were no statistical differences in these metrics between international or local partners (Figure 5B; Supplement Table S2). SIMS data also supported strong service delivery quality for OVC, adolescent girls and young women, and KP programming (Figure 7B); this is especially notable because local partners providing these services in FY2019 and FY2020 were often new local prime partners.^20^ Local partners struggled with achieving PrEP targets in FY20 (Figure 2B, 5), perhaps because of the substantial increase in targets for South African local partners between FY2019 and FY2020 (Figure 4). Both international and local partners struggled with achieving VMMC in FY2020 (Figures 2B and 5), potentially because of changes in eligibility priorities for VMMC target achievement, as well as a pause on non-emergency procedures during the COVID-19 pandemic.^21^ There was no statistical difference in the distribution of VMMC achievement for either FY2019 or FY2020 between international or local partners (Supplement Tables S1 and S2), but very small sample sizes for local partner VMMC mean that this metric should be reevaluated at the local partner cohort continues to grow.

### Limitations and Future Directions

Although a primary goal of this article was to assess local partner performance at a global scale, making a global comparison has limitations and potentially masks individual country or partner impacts on the performance against targets. One key limitation for evaluating percent achievement is that the annual target-setting process is highly fluid and often influenced by a country’s political, epidemiological, and budgetary context. For additional context, we provided OU and individual-level comparisons (Figures 5 and 6; Supplement Tables S1–S3). However, there were several important partner-level factors that we were not able to control for in this analysis, including how long a partner had been serving in a prime capacity, organizational size, organizational connections, or whether an organization is operating in a rural or urban setting. Unfortunately, PEPFAR does not collect data on the type of organization (with the exception of government partners versus non-government partners) and only recently began collecting staffing data (beginning in FY2021 after the analysis period for this article). Future work could involve interfacing with partners directly to collect this organization-specific information and control for these factors to help explain performance outcomes. This analysis also has a limited sample size (Figures 5 and 6; Supplement Tables S1–S3). Although the local partner cohort is growing,^20^ not all partners report on all targets, which means that comparing international vs. local partner performance still lacks statistical power, especially for certain indicators (number of males circumcised through the VMMC program in particular). Because of this smaller sample size and reliance on a non-parametric statistical test, a lack of significance should not be considered conclusive of a lack of difference in the medians of the distributions between local and international partners.

Like MER target setting, the selection of sites to be assessed for SIMS is heavily dependent on country context, and sites are not randomly distributed (Supplement Figure). In addition, COVID-19 lockdowns and restrictions on travel significantly limited USAID’s ability to conduct SIMS visits in Quarter 3 and Quarter 4 of FY2020, with only 7 of the 23 LTS countries completing more than 50% of their assessments for local partners in FY2020. Because of this site selection variability, we focused on technical areas and CEEs with larger sample sizes, but results should still be interpreted cautiously in terms of their generalizability to all local partner programs. As highlighted in the results, the proportion of SIMS site visits did not align proportionally with OU-level or partner-level targets, which limits their generalizability. With only 2 years of performance and quality of service data to date, continually reviewing results will be critical to ensure that the transition is monitored and progress is sustained.

These findings for both international and local partners should be contextualized within the disruption of the COVID-19 pandemic. Pandemic-related lockdowns restricted the ability of many PLHIV to receive services at health care facilities and hindered community-based services, such as OVC, KP, or VMMC programs. While national-level policies encouraged multi-month ART dispensing and uptake of PrEP, implementing partners had to adapt their programming to minimize the disruption of services. It will be important to monitor the effects of ongoing restrictions and lockdowns on implementing partners in PEPFAR-funded OUs.

Lastly, while this analysis focuses on implementing partners, it is important to acknowledge that all partners are operating within national health systems and specific country contexts. Policies, limitations, and capacities will inherently vary by country. Future studies could consider a cohort study of site-level comparisons of implementing partners that have and have not transitioned, particularly to identify countries where localization efforts have been very successful. Future research could also explore the financial performance of local and international partners, the effectiveness of activities occurring at a national level (not measured by MER targets), and the performance of new versus established local partners.

## CONCLUSION

While localization is a commonly expressed value in the global development space, there are also coexisting concerns about rapid transitions negatively affecting the scale-up and ongoing implementation of HIV/AIDS services.^5^ We examined USAID’s PEPFAR Local Partner Transition and found that while local partners are still working towards building their treatment cohort size to match targets, they performed and delivered quality of service that was comparable to international partners across several MER and calculated indicators—particularly along the clinical cascade once patients had been identified. This held true even though about half of the local partners were serving as prime partners for the first time and COVID-19 impacted the ability of all partners to implement programs. Given the shorter timeline of performance evaluation, the smaller sample size of individual partners, and the inherent limitations of the performance and quality of service metrics discussed above, these findings should be interpreted cautiously and revisited as more data become available.

## Supplementary Material

GHSP-D-22-00337-supplement.pdf

## Notes

**Peer Reviewed**

**First published online:** June 9, 2023.
