# Supplementary material for: An Evaluation of Local Implementing Partner Performance During the First 2 Years of the USAID/PEPFAR Transition
Source: Glob Health Sci Pract. 2023 Jun 21;11(3):e2200337. doi: 10.9745/GHSP-D-22-00337 (PMC10285723; doi:10.9745/GHSP-D-22-00337)
Supplement: GHSP-D-22-00337-supplement.pdf [file GHSP-D-22-00337-supplement.pdf]

Supplement to: White LA, Avery L, Bonanno L, et al. An evaluation of local implementing partner performance during the first 2 years of the USAID/PEPFAR transition. *Glob Health Sci Pract*. 2023;11(3):e2200337. https://doi.org/10.9745/GHSP-D-22-00337

Expanded definitions of key terms

Local partner

According to COP guidance, an entity may be considered local if it meets the following criteria: 1) EITHER must be incorporated or legally organized under the laws of, and have its principal place of business in, the country served by the PEPFAR program with which the entity is or may become involved OR must exist in the region where the entities funded PEPFAR program are being implemented and 2) EITHER must be at 75% for FY 2018 beneficially owned by individuals who are citizens or lawfully admitted permanent residents of that same country OR at least 75% for FY 2018 of the entity's staff must be citizens or lawfully admitted permanent residents of that same country, and at least 75% for FY 2018 of the entity's senior staff [1].

Long term strategy (LTS) countries

The LTS countries previously were Botswana, Burundi, Cameroon, Côte d'Ivoire, Democratic Republic of the Congo, eSwatini, Ethiopia, Haiti, Lesotho, Kenya, Malawi, Mozambique, Namibia, Nigeria, Rwanda, South Africa, South Sudan, Tanzania, Ukraine, Uganda, Vietnam, Zambia, and Zimbabwe. USAID prioritized these 23 countries at the start of the PEPFAR Local Partner Transition, given their larger distribution of budget and targets.

Additional description of methods:

Budget: funding amounts and percentages

Funding calculations include 23 LTS OUs only. Data include Central TLS and Acceleration 20 funding in all years, but regional partners only in FY21 per COP Guidance. Percentages exclude Global Health Supply Chain (GHSC) Procurement and Supply Chain Management, GHSC Rapid Test Kits, and Management and Operations. FY17-FY20 funding data are derived from FACTS Info/Standard COP Matrix Report. FY21 funding data are derived from the FACTS Info/Standard COP Matrix Report and kept current through manual tracking. All funding is based on Facts Info (via COP Matrix), except COP21 LP funding level (based on SBU/LP work plans).

"Acceleration 20" funding and Central Initiatives (Central TLS) are included in all FYs.

"Acceleration 20 funding" refers to funding allocated to Mission teams by State/Global AIDS Coordinator (S/GAC) for specific implementation needs and is separate from Mission's COP funding. Central Initiative Funding (also referred to as Central TLS), are central funds allocated to Missions at the discretion of the Global AIDS Coordinator and Health Diplomacy.

Key indicator definitions per "MER Indicator Reference Guide v.2.5" [2]

- **HTS_TST_POS:** "Number of individuals who received HIV Testing Services (HTS)" and received positive test results. Reported quarterly.
- **TX_NEW:** "Number of adults and children newly enrolled on antiretroviral therapy (ART)." Reported quarterly.
- **TX_CURR:** "Number of adults and children currently receiving antiretroviral therapy (ART)." Reported quarterly.
- **PrEP_NEW:** "Number of individuals who were newly enrolled on oral antiretroviral pre-exposure prophylaxis (PrEP) to prevent HIV infection in the reporting period." Reported quarterly.
- **KP_PREV:** "Number of key populations reached with individual and/or small group-level HIV prevention interventions designed for the target population." Reported semi-annually.
- **OVC_SERV:** "Number of beneficiaries served by PEPFAR OVC programs for children and families affected by HIV." Reported semi-annually.
- **VMMC_CIRC:** "Number of males circumcised as part of the voluntary medical male circumcision (VMMC) for HIV prevention program within the reporting period." Reported semi-annually.

Calculated indicator definitions per "MER Indicator Reference Guide v.2.5" [2]

- **Testing yield:** "Within a testing program, the percentage of positives found out of those who were tested and received their test results. Yield can be used for general testing as well as targeted testing for PMTCT, TB, etc." General testing yield: HTS_TST_POS/(HTS_TST_POS + HTS_TST_NEG)
- **Linkage to treatment proxy:** "Estimate of the percentage of people who test HIV positive and are linked to treatment. This metric is not calculated at the individual level." Calculation: TX_NEW/HTS_TST_POS
- **Viral load coverage:** "Comparison of the denominator for this indicator [TX_PVLS] with the result for TX_CURR from 6 months earlier (i.e., two quarters prior) can be used to crudely estimate VL testing coverage supported by PEPFAR."
- **Viral load suppression coverage:** "Percentage of ART patients with a suppressed viral load (VL) result (<1000 copies/ml) documented in the medical or laboratory records/laboratory information systems (LIS) within the past 12 months." Calculation: TX_PVLS numerator/TX_PVLS denominator

SIMS and CEEs

The scores on SIMS are coded as "Needs Urgent Remediation", "Needs Improvement", and "Meets Standard". Each year the USG teams prioritize sites to be visited and conduct follow up visits to any partners with "Needs Urgent Remediation" or "Needs Improvement" scores within six months.

Each set category combines a group of relevant CEEs [3]:

"*HIV Testing*" includes CEEs on compliance with national testing algorithm and strategy, quality assurance of HIV testing services, HTS linkage to HIV care and treatment at the site level, site level HIV proficiency testing, HTS safety measures at the site, confidentiality of HIV testing services at the site, HIV self-testing, index testing training and supportive supervision, monitoring adverse events, secure handling and storage of index testing data, and intimate partner violence risk assessment and support.

"*Care and Treatment"* includes CEEs on retesting for verification before/at ART initiation, patient tracking-ART patients, rapid ART initiation, viral load access and monitoring, management of high viral load, appointment spacing and multi-month drug dispensing, partner services, routine HIV testing of children of adult patients, TB screening, TB preventive treatment (TPT)/Isoniazid preventive therapy (IPT), Cotrimoxazole (CTX), TB diagnostic evaluation cascade, community-based linkage and retention support services, service referral and linkage system, family planning/HIV integration service delivery, community-based delivery of family planning services, and cervical cancer screening capacity.

"*AGYW, GBV, and OVC*" includes CEEs on capacity to provide post-violence care services, availability of post-violence care services, gender norms, case management services, case management workforce strengthening, preventing HIV in girls, services to support HIV testing for OVC, services to support HIV treatment linkage, retention, and viral suppression for OVC.

References:

1. U.S. Department of State. PEPFAR 2021 Country and Regional Operational Plan (COP/ROP) Guidance for all PEPFAR Countries [Internet]. 2021 Feb. Available from: https://www.state.gov/wp-content/uploads/2021/02/PEPFAR-COP21-Guidance-Final.pdf

2. President's Emergency Plan for AIDS Relief. Monitoring, Evaluation, and Reporting Indicator Reference Guide: MER 2.0 (Version 2.5) [Internet]. 2020 Sep. Available from: https://datim.zendesk.com/hc/en-us/articles/360000084446-MER-Indicator-Reference-Guides

3. DATIM. FY21 SIMS 4.1 Materials [Internet]. 2021. Available from: https://datim.zendesk.com/hc/en-us/articles/360048154711-FY21-SIMS-4-1-Materials

Supplement to: White LA, Avery L, Bonanno L, et al. An evaluation of local implementing partner performance during the first 2 years of the USAID/PEPFAR transition. *Glob Health Sci Pract*. 2023;11(3):e2200337. https://doi.org/10.9745/GHSP-D-22-00337

Supplement Figures

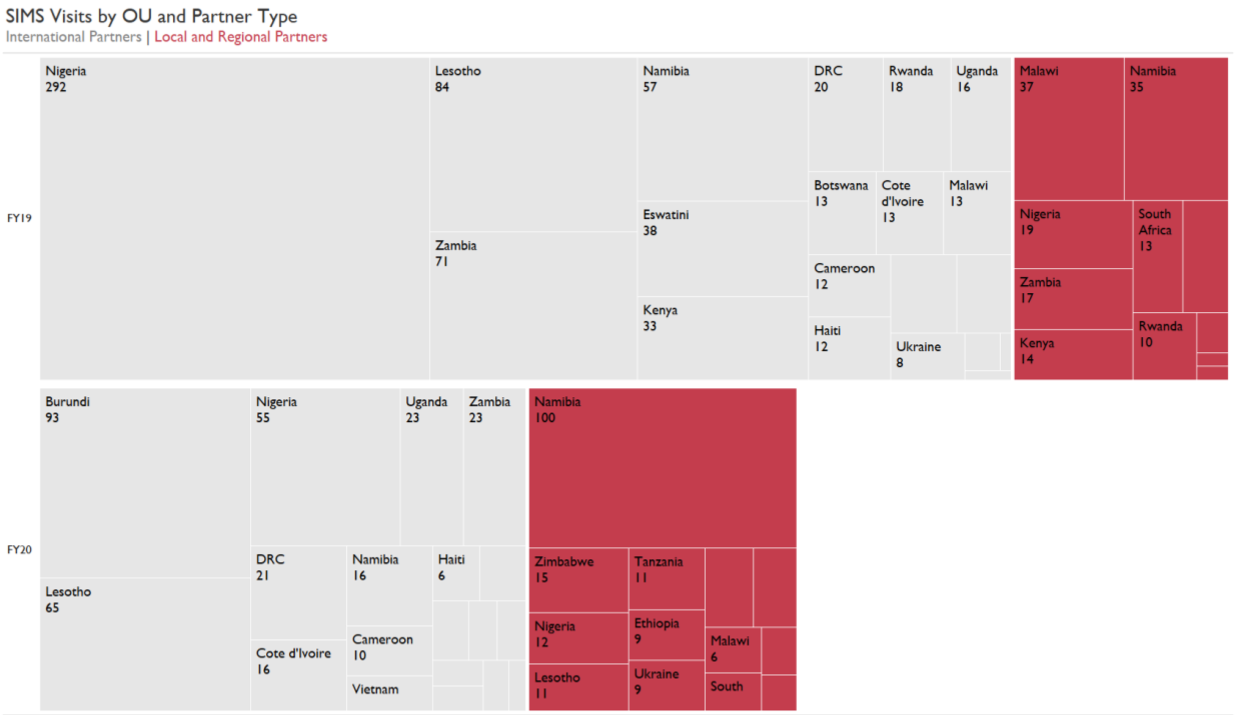

Supplement Figure. Tree map depicting the number of SIMS visits broken down by OU for international and local partners in FY19 and FY20. Cell size is proportional to the number of SIMS visits in each OU.

Supplement to: White LA, Avery L, Bonanno L, et al. An evaluation of local implementing partner performance during the first 2 years of the USAID/PEPFAR transition. *Glob Health Sci Pract*. 2023;11(3):e2200337. https://doi.org/10.9745/GHSP-D-22-00337

Table S1. Mean and median percent achievement rates of operating units (OUs) disaggregated by fiscal year (FY), MER indicator, and partner type. For FY19, TX_CURR Botswana was a significant outlier with a percent achievement of 9900%, which skewed the overall mean. The reported p-values and test statistics are from a two-sided Wilcoxon rank sum test with continuity correction with the alternative hypothesis being the true location shift is not equal to 0.

MER Indicator	Fiscal Year (FY)	Partner Type	Number of Partners	Mean percent achievement (%)	Median percent achievement (%)	W-statistic and p-value
HTS_TST_POS	FY19	International	22	103.44	93.07	W = 135; p = 0.32
	FY19	Local	10	89.38	80.58	
	FY20	International	21	93.27	77.83	W = 168; p = 0.77
	FY20	Local	17	108.83	87.87	
TX_NEW	FY19	International	18	97.76	92.05	W = 60; p = 0.53
	FY19	Local	8	201.8	109.78	
	FY20	International	19	96.4	81.94	W = 101; p = 0.4
	FY20	Local	13	113.08	114.42	
TX_CURR	FY19	International	18	630.45	87.13	W = 65; p =0.72
	FY19	Local	8	110.03	90.56	
	FY20	International	19	99.8	90.85	W= 117; p= 0.82
	FY20	Local	13	96.51	91.6	
OVC_SERV	FY19	International	19	101.08	99.69	W = 56; p =0.57
	FY19	Local	7	110.98	101.8	
	FY20	International	17	120.88	102.56	W = 120; p = 0.71
	FY20	Local	13	101.52	105.59	
KP_PREV	FY19	International	19	113.72	118.09	W = 40; p = 0.91
	FY19	Local	4	101.71	109.02	
	FY20	International	18	129.07	115.28	W = 110; p =0.64
	FY20	Local	11	133.78	114.57	

**MER Indicator**	**Fiscal Year (FY)**	**Partner Type**	**Number of Partners**	**Mean percent achievement (%)**	**Median percent achievement (%)**	**W-statistic and p-value**
PrEP_NEW	FY19	International	14	125.12	93.67	W = 15; p = 0.19
		Local	4	245.12	114.99	
	FY20	International	16	185.04	153.54	W = 105; p = 0.42
		Local	11	212.00	82.85	
VMMC_CIRC	FY19	International	10	93.67	90.91	*not reported; $n_2 < 3$
		Local	2	78.46	78.46	
	FY20	International	10	59.64	55.24	W= 21; p = 0.95
		Local	4	63.22	55.4	

Supplement to: White LA, Avery L, Bonanno L, et al. An evaluation of local implementing partner performance during the first 2 years of the USAID/PEPFAR transition. *Glob Health Sci Pract*. 2023;11(3):e2200337. https://doi.org/10.9745/GHSP-D-22-00337

Table S2. Mean and median percent achievement rates of individual partners disaggregated by fiscal year (FY), MER indicator, and partner type. The reported p-values and test statistics are from a two-sided Wilcoxon rank sum test with continuity correction with the alternative hypothesis being the true location shift is not equal to 0.

MER Indicator	Fiscal Year (FY)	Partner Type	Number of Partners	Mean percent achievement (%)	Median percent achievement (%)	W-statistic and p-value
HTS_TST_POS	FY19	International	58	118.98	101.28	W = 846; p = 0.13
		Local	24	108.21	79.76	
	FY20	International	51	462.92	77.83	W = 895; p = 0.99
		Local	35	690.74	72.71	
TX_NEW	FY19	International	33	108.71	100.64	W = 352; p = 0.47
		Local	19	137.42	77.44	
	FY20	International	34	109.93	84.04	W= 475; p = 0.63
		Local	26	106.84	69.46	
TX_CURR	FY19	International	32	93	89.94	W = 317; p = 0.57
		Local	18	97.63	86.99	
	FY20	International	33	104.16	93.09	W = 435; p = 0.36
		Local	23	101.74	88.21	
OVC_SERV	FY19	International	28	102.42	99.83	W = 208; p = 0.33
		Local	18	114.23	103.01	
	FY20	International	22	207.04	101.24	W = 364; p = 0.69
		Local	31	108.38	104.26	
KP_PREV	FY19	International	26	110.71	112.79	W = 58; p = 0.74
		Local	5	107.17	127.37	
	FY20	International	26	176.61	114.07	W =241; p = 0.4
		Local	16	124.4	106.91	

PrEP_NEW	FY19	International	25	143.61	100.00	W = 110; p = 0.94
		Local	9	169.66	88.07	
	FY20	International	28	253.00	145.23	W = 348; p = 0.16
		Local	20	187.57	61.64	
VMMC_CIRC	FY19	International	18	97.37	98.65	W = 41; p = 0.18
		Local	3	77.39	77.6	
	FY20	International	19	68.67	70.19	W = 42; p = 0.78
		Local	4	63.22	55.4	

Supplement to: White LA, Avery L, Bonanno L, et al. An evaluation of local implementing partner performance during the first 2 years of the USAID/PEPFAR transition. *Glob Health Sci Pract*. 2023;11(3):e2200337. https://doi.org/10.9745/GHSP-D-22-00337

Table S3. Summary statistics of individual partners' calculated indicator rates disaggregated by partner type and fiscal year. The statistical test performed was a Wilcoxon rank sum test with continuity correction with the alternative hypothesis being the true location shift is not equal to 0.

Calculated Indicator	Fiscal year (FY)	Partner type	Number of partners	Mean (%)	Median (%)	W-statistic, p-value, and empirical power
Test positivity	FY19	International	63	6.96	4.5	W = 960; p= 0.51
		Local	28	5.33	4.6	
	FY20	International	57	7.61	4.9	W = 985; p= 0.46
		Local	38	6.67	5.85	
Linkage	FY19	International	35	97.68	91.5	W = 296.5; p = 0.52; empirical power = 0.097
		Local	19	92.68	93.2	
	FY20	International	37	95.82	94.5	W = 488.5; p= 0.7; empirical power= 0.052
		Local	28	92.65	94.9	
Viral load coverage (VLC)	FY19	International	31	81.55	86.2	W =270; p =0.63
		Local	16	76.95	82.1	
	FY20	International	31	84.47	84.8	W=458.5; p= 0.08
		Local	23	70.71	72.8	
Viral load suppression (VLS)	FY19	International	31	87.51	89.3	W= 193; p=0.08
		Local	18	90.9	91.15	
	FY20	International	32	91.71	92.45	W= 395.5; p = 0.95
		Local	25	92.26	92.5	
